# Three-dimensional analysis of posttreatment tooth movements despite bonded retainers: part II—lower jaw

**DOI:** 10.1007/s00056-024-00546-x

**Published:** 2024-08-23

**Authors:** Katharina Klaus, Tobias Kleinert, Sabine Ruf

**Affiliations:** 1 Department of Orthodontics, Justus Liebig University Giessen, Schlangenzahl 14, 35392 Giessen, Germany; 2 Private Practice, Balingen, Germany

**Keywords:** Bonded lingual retainer, Mandibular bonded retainer, Orthodontic retention, Retainer complications, Posttreatment malocclusion, Festsitzende Lingualretainer, Unterkiefer-Kleberetainer, Kieferorthopädische Retention, Retainerkomplikationen, Fehlstellung nach Behandlung

## Abstract

**Purpose:**

Complications of bonded lingual retainers in terms of unexpected tooth movements have been reported with increasing frequency during the last decade, but the vast majority of the literature comprises case reports. The purpose of the present retrospective case–control study was to analyze the amount and direction of unwanted tooth movements despite lower bonded retainers, to analyze possible predisposing pretreatment- and treatment-related factors, and to seek for movement thresholds which could enhance the rapid and objective identification of critical cases.

**Methods:**

Plaster casts of 1026 patients who completed orthodontic treatment and a subsequent retention phase of 2 years were screened for unintentional tooth movements. The study group comprised 39 patients with tooth movements in the lower jaw, while 39 randomly selected patients without visible tooth movements served as the control group. For all patients, plaster casts after debonding of multibracket appliances (T1) and after supervised retention (T2) were digitized, and a three-dimensional (3D) digital superimposition based on the best fit of premolars and molars was undertaken. Thereafter, translational as well as rotational movements were measured in all three planes of space. Pretreatment- and treatment-related factors of the study and control groups were compared. A severity classification based on rotational movement thresholds was applied to seek a critical amount of translational movements.

**Results:**

The mean translational movements ranged between 0 and 0.4 mm and the average rotational movements between 0 and 1.6°. Large individual movements up to 1.9 mm translation and 16° rotation were seen. A twist-effect with opposite movements of the canines along the Y‑axis could be confirmed. Compared to the control group, patients of the study group had a smaller intercanine distance at all timepoints. In addition, study group patients presented a slightly larger intercanine expansion during treatment and were more often affected by retainer bonding site detachments. Applying the severity classification based on rotational thresholds, translational movements of 0.5–1.0 mm along the X‑ and Y‑axis could serve as a critical threshold. It can be assumed that extrusive translational movements along the Z‑axis seem to be of specific nature and perhaps do not reflect a retainer complication in terms of unwanted tooth movements.

**Conclusions:**

Patients with a larger intercanine distance after active treatment and those with more frequent retainer bonding site detachments could be at higher risk for unwanted tooth movements during fixed retention. Sagittal and transverse movements of 0.5–1.0 mm should sensitize the practitioner for further measures.

## Abstract (de)

**Zielsetzung:**

Komplikationen festsitzender lingualer Retainer im Sinne unerwünschter Zahnbewegungen wurden während der vergangenen 10 Jahre immer häufiger berichtet, jedoch handelt es sich bei der überwiegenden Mehrheit der Literatur um Fallberichte. Die Zielsetzung der vorliegenden retrospektiven Fall-Kontroll-Studie war, das Ausmaß und die Richtung unerwünschter Zahnbewegungen zu untersuchen, mögliche prädisponierende prätherapeutische und therapeutische Faktoren zu analysieren sowie Bewegungsgrenzwerte zur schnelleren und objektiveren Aufdeckung von Risikopatienten zu identifizieren.

**Material und Methode:**

Studienmodelle von 1026 Patienten, welche ihre aktive kieferorthopädische Behandlung inklusive zweijähriger Retention mittels festsitzender Retainer abgeschlossen hatten, wurden hinsichtlich unerwünschter Zahnbewegungen im Bereich des Retainers gesichtet. Die Studiengruppe umfasste 39 Patienten mit Zahnbewegungen im Unterkiefer, 39 zufällig ausgewählte Patienten ohne sichtbare Zahnbewegungen dienten als Kontrollgruppe. Die Modelle aller Patienten zum Zeitpunkt der Entfernung der Multibracketapparatur (T1) sowie nach überwachter Retentionszeit (T2) wurden digitalisiert und mittels Best-fit-Überlagerung von Prämolaren und Molaren superponiert. Anschließend wurden die translatorischen und rotatorischen Zahnbewegungen in allen drei Dimensionen vermessen. Prätherapeutische und therapeutische Variablen der Studien- und Kontrollgruppe wurden verglichen. Eine Klassifikation des Schweregrads unerwünschter Zahnbewegungen basierend auf rotatorischen Grenzwerten wurde angewendet, um ein kritisches Ausmaß translatorischer Zahnbewegungen zu identifizieren.

**Ergebnisse:**

Die durchschnittlichen translatorischen Bewegungen lagen zwischen 0 und 0,4 mm, während die mittleren rotatorischen Bewegungen zwischen 0 und 1,6° betrugen. Große individuelle Zahnbewegungen bis zu 1,9 mm Translation und 15,9° Rotation wurden erfasst. Ein Twist-Effekt mit gegensätzlichen Bewegungsmaxima der unteren Eckzähne entlang der Y‑Achse konnte identifiziert werden. Verglichen mit der Kontrollgruppe, wiesen die Patienten der Studiengruppe zu allen Zeitpunkten eine geringere intercanine Distanz auf. Außerdem zeigte sich in der Studiengruppe eine etwas größere intercanine Expansion während der Behandlung sowie häufigere Klebestellenverluste während der Retentionsphase. Unter Anwendung der rotatorischen Grenzwerte könnten translatorische Bewegungen entlang der X‑ und Y‑Achse im Bereich zwischen 0,5 und 1 mm als kritische Grenzwerte dienen. Die Vermutung liegt nahe, dass extrusive translatorische Bewegungen entlang der Z‑Achse eine Sonderrolle spielen und unter Umständen keine Retainerkomplikation im Sinne einer unerwünschten Zahnbewegungen darstellen.

**Schlussfolgerungen:**

Patienten mit einer größeren intercaninen Distanz nach der aktiven Behandlung beziehungsweise solche mit vermehrten Klebestellenverlusten ihrer Retainer könnten einem höheren Risiko für unerwünschte Zahnbewegungen während festsitzender Retention ausgesetzt sein. Sagittale und transversale Bewegungen zwischen 0,5 und 1,0 mm sollten den Behandler für weitere Maßnahmen sensibilisieren.

## Introduction

Since their introduction in the 1970s, fixed lingual retainers have become the gold standard of retention method in the lower jaw [1, 18, 22, 23, 26, 31, 35]. Compared to removable retainers, bonded lingual retainers are independent of patient cooperation, which is one of their greatest advantages [23, 23, 26]. Nevertheless, retention with fixed retainers is not unproblematic—complications like bonding site detachments and wire breakages have been described frequently [15, 20, 27, 36]. But during the last 15 years, a new kind of complication has been increasingly noted by the orthodontic community: Katsaros et al. were the first to describe unexpected tooth movements in terms of either a torque difference between two adjacent incisors (X-effect) or an increased buccal inclination of one mandibular canine despite of seemingly intact flexible spiral wire retainers bonded to all six anterior teeth in the mandible [11]. Kucera and Marek [17] additionally reported the twist-effect: an increased opposite inclination of both mandibular canines [17]. Such movements are classified as newly developed malocclusion rather than relapse, because they show no similarities to the pretreatment malocclusion [11, 17, 25]. A recently published systematic review used the term “wire syndrome” to sum up those complications and analyzed 20 articles. However, the authors revealed a high risk of bias because 16 of the articles were case reports/case series [7]. Only a few retrospective studies [12, 17, 34] dealt with this topic and tried to identify risk factors by comparing affected and unaffected subjects. They found prevalence values ranging from 1.1 to 43.3% of the patients. Possible predisposing factors were the following: a younger age at debonding, larger pretreatment mandibular plane angles, an increased pretreatment incisor inclination [17], larger amounts of intercanine expansion and overjet reduction during treatment [34], pretreatment oral dysfunctions or habits, as well as pre- and posttreatment overbite with lack of interincisal contact [12]. Nevertheless, there is no consensus about the possible risk factors and all in all, the etiology of posttreatment tooth movements despite bonded retainers is still unknown today [7].

Taking the ongoing digitalization of dentistry into account, a future vision for further prospective studies and clinical routine could be the superimposition of intraoral scans to identify patients at risk by detecting already small tooth movements before they become clinically visible and need retreatment. Therefore, a definition of tooth movement thresholds which reflect the visible change in tooth positions is mandatory. Of the above-mentioned retrospective studies, Wolf et al. [34] analyzed their patients by superimposing and measuring digitized plaster casts and categorized them into severity groups depending on the amount of rotational tooth position changes. As arbitrary thresholds, rotations of 0–5° were considered as stable, 5–9° as moderately affected, and rotations larger than 9° as severely affected [34]. Up to date, the digital measurement of single tooth movements after superimposition of digital casts is still a time-consuming procedure and not suitable for clinical routine. A faster alternative is the visualization of surface comparisons based on a best-fit superimposition in terms of a so-called heatmap or color-coded distance map, which can be undertaken by all relevant computer-aided design (CAD) software and even some intraoral scanner software and is recently used in orthodontic research for different purposes [5, 8, 10, 12, 16, 24, 30]. However, those distance maps are based on a metric scale and visualize the deviations between two models in millimeters, so thresholds based on translational rather than rotational tooth movements would be desirable.

Therefore, the aims of the present retrospective case–control study were (1) to investigate the amount and direction of unwanted tooth movements despite lower bonded retainers in situ, (2) to analyze possible predisposing pretreatment and treatment-related factors, and (3) to seek for translational movement thresholds by applying the rotational measurement thresholds introduced by Wolf et al. [34]. This is the second part of a study dealing with unwanted posttreatment tooth movements despite bonded retainers in situ, the first part described such complications for the upper jaw [13].

## Materials and methods

**Fig. 1:**
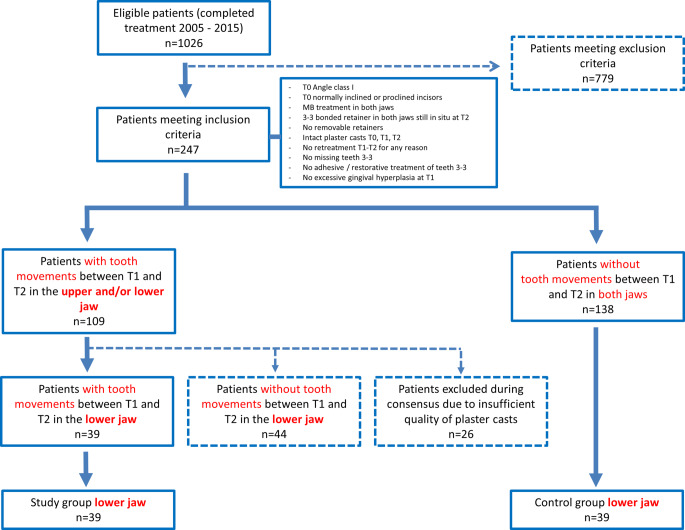
Flowchart of study population Flussdiagramm zur Studienpopulation

The records of all patients who completed active orthodontic treatment and a subsequent supervised retention period of approximately 2 years between 2005 and 2015 at the Department of Orthodontics of the Justus Liebig University Giessen, Germany, were screened. A detailed description of the inclusion and exclusion criteria as well as ethical approval can be found in part I of this study [13], a flowchart illustrating the study population is given in Fig. 1.

In addition, the retention protocol and the methods used for case identification, model digitization, digital superimposition, and measurement of tooth movements have been fully described elsewhere [12, 13]. Therefore, only the main information is summarized hereafter. All retainers were manufactured of 0.018 inch 6‑strand coaxial wire (Dentaflex, Dentaurum, Ispringen, Germany), bonded by the orthodontists and residents of the department according to the direct bonding method described by Zachrisson and Büyükyilmaz [37] using Transbond XT or Transbond LR (3M Unitek, Monrovia, CA, USA). Regularly control visits were scheduled. In cases of retainer debondings or wire breakages, patients were advised to make an emergency repair appointment as soon as possible.

**Fig. 2:**
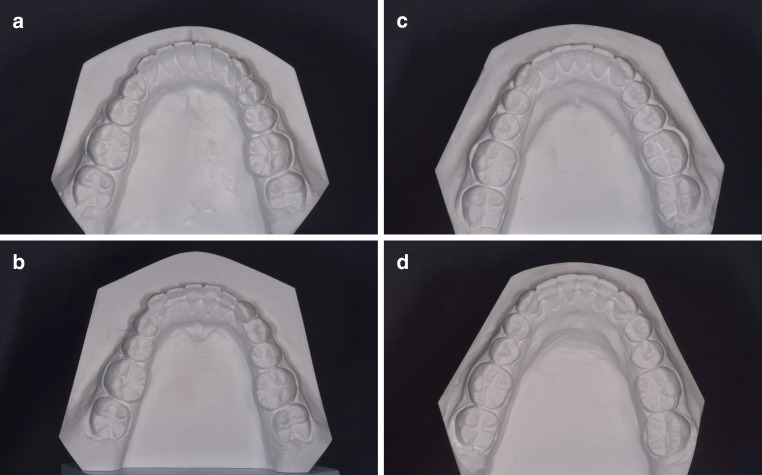
Exemplary cases of visual inspection for assignment to the study (**a**, **b**) and control (**c**, **d**) groups. **a** T1 model of study group patient. **b** T2 model of the same patient with twist-effect. **c** T1 model of control group patient. **d** T2 model of the same patient Exemplarische Fälle visueller Inspektion für die Zuteilung zur Studien- (**a**, **b**) bzw. zur Kontrollgruppe (**c**, **d**). **a** T1-Modell eines Studiengruppenpatienten. **b** T2-Modell desselben Patienten mit Twist-Effekt. **c** T1-Modell eines Kontrollgruppenpatienten. **d** T2-Modell desselben Patienten

From the patients meeting the inclusion criteria, all plaster casts at T1 (debonding of multibracket appliance) and at T2 (after supervised retention phase) were visually inspected by one operator (T.K.) comparing the tooth positions of the canines as well as of the lateral and central incisors between the two time points. All suspicious casts were additionally judged by two experienced orthodontists (K.K., S.R.). To reach consensus in uncertain cases, the incisal edges and marginal ridges were colored using a soft pencil. All patients for whom all authors were in agreement regarding tooth movements in the lower jaw between T1 and T2 were assigned to the study group, while an equal number of patients without visible tooth movements in both jaws were randomly chosen for the control group. Figure 2 shows plaster casts of two exemplary patients who were assigned to the study or the control group, respectively, based on visual inspection.

**Fig. 3:**
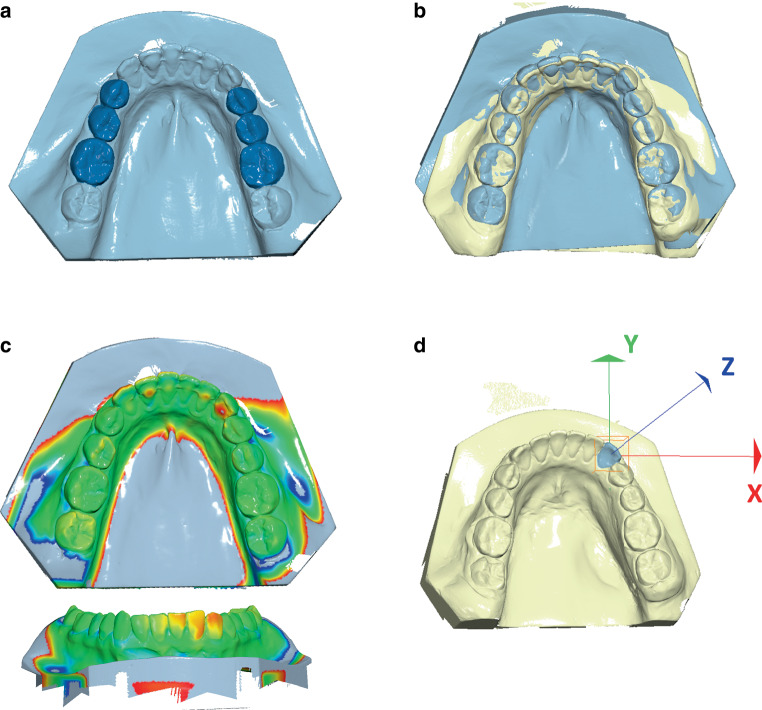
**a** The reference area used for superimposition of digital models. **b** Superimposed models from time points T1 (after multibracket appliance removal, *yellow*) and T2 (after 2 years of supervised fixed retention, *light blue*). **c** Colormap indicating the deviations based on the T2 model ranging between +1.5 mm (*red*) and −1.5 mm (*blue*). **d** Orientation of axes for measurement of tooth movements **a** Referenzbereich für die digitale Modellüberlagerung. **b** Überlagerte Modelle der Zeitpunkte T1 (nach Entfernung der Multibracketapparatur, *gelb*) und T2 (nach zwei Jahren überwachter festsitzender Retention, *hellblau*). **c** Farbige Abweichungsdarstellung auf Basis der T2-Modelle, Abweichungen reichen von +1,5 mm (*rot*) bis −1,5 mm (*blau*). **d** Orientierung der Achsen für die Vermessung der Zahnbewegungen

All lower plaster casts of the study and control groups (T1, T2) were digitized using a desktop scanner (OrthoXScan, Dentaurum, Ispringen, Germany) and saved as Standard Tesselation Language (STL) files. The digitized casts of T1 and T2 were imported into the Viewbox 4 software (Version 4.1.0.6 BETA, dHAL software, Kifissia, Greece) and superimposed using a best-fit matching of the first molars and the premolars. The latter was necessary due to the lack of stable reference structures in the mandible (Fig. 3a). The software uses a best-fit superimposition based on the implementation of the iterative closest point algorithm (ICP) [2] according to settings described elsewhere ([13, 32, 33]; Fig. 3b). Afterwards, a distance map was created, color-coding the deviations between the superimposed models (red: +1.5 mm to blue: −1.5 mm, Fig. 3c). Furthermore, the movements of all teeth showing a color deviation were measured in the three planes of space, as described elsewhere [13, 32, 33]. The X‑axis (red) was defined parallel to the occlusal plane and perpendicular to the midline (lateral translational movements; positive: right, negative: left), the Y‑axis (green) was defined parallel to the occlusal plane and parallel to the midline (anterior–posterior translational movements; positive: protrusion, negative: retrusion) and the Z‑axis (blue) was defined perpendicular to the occlusal plane (vertical translational movements; positive: extrusion, negative: intrusion). Rotational movements around the X‑axis (inclination changes in anteroposterior direction; positive: proclination, negative: retroclination), around the Y‑axis (lateral inclination; positive: right, negative: left), and around the Z‑axis (rotational changes; positive: counterclockwise, negative: clockwise) were also measured (Fig. 3d). Translational changes were measured in millimeters (mm), while rotational movements were recorded in degrees (°).

The following data were obtained from the patients’ records:

- Sex,
- Age of the patients pretreatment (T0), after debonding of the multibracket appliance (T1), and after a supervised retention phase approximately two years later (T2),
- Existence of pretreatment habits (sucking habit, lip or tongue dysfunction, tongue thrust swallowing, bruxism, mouth breathing),
- T0 cephalometric mandibular plane angle (ML/NSL),
- T0 cephalometric lower incisor inclination angle (IiL/NB),
- T0 cephalometric lower incisor inclination to A‑Pogonion line (Ii-APo),
- extraction of premolars during active treatment,
- T0, T1, and T2 overjet,
- T0, T1, and T2 incisal relationship (interincisal contact, incisal overlap without interincisal contact, open bite),
- T0, T1, and T2 intercanine distance,
- Retainer bonding site detachments between T1 and T2, and
- Retainer wire breakages between T1 and T2.

### Statistical analysis

In accordance with part I of this study [13], statistical analysis was performed calculating intraclass correlation coefficients (ICCs) to check the intraobserver reliability of repeated digital superimpositions as well as of measurements of tooth movements. Furthermore, study and control groups were compared using descriptive statistics; the Χ^2^ test and the exact Fisher test were used for comparison of categorial variables. After testing for normal distribution (Kolmogorov–Smirnov test, Shapiro–Wilk test), the Mann–Whitney U test and the T‑test for independent samples were applied for comparison of numeric variables. Statistics were carried out by a certified medical biostatistician using the software SPSS for windows, version 25.0 (IBM Corp., Armonk, NY, USA). The level of significance was set at *p* < 0.05.

## Results

The final study group comprised 39 patients (15 male, 24 female; mean age at T0: 12.82 ± 1.92 years). Active orthodontic treatment (T0–T1) of the study group patients lasted 3.01 ± 1.07 years, while the duration of supervised retention was 2.05 ± 0.38 years. The control group also comprised 39 patients with the same gender distribution as the study group and an average pretreatment age of 12.22 ± 1.87, a mean active treatment time of 3.17 ± 1.23 years and a supervised retention period of 2.08 ± 0.32 years. No statistically significant differences between the groups were found for age, treatment, and retention time.

### Tooth movements

An excellent intraobserver reliability was indicated by the intraclass correlation coefficients (ICC) for repeated digital superimpositions (0.980) as well as for the measurement of tooth movements (0.982).

The visually recognized tooth movements of the study group patients were confirmed during 3D superimposition. The highest movement prevalence was seen for tooth 41 (76.9%), followed by 31 (69.2%), both canines (66.7% each) and the lateral incisors (42: 64.1%, 32: 61.5%). Nevertheless, even in the control group, 3D superimposition showed tooth movements with prevalence values ranging from 43.6% at the central incisors to 59% at tooth 32, which were not detected by visual inspection of the plaster casts. However, the amount of absolute tooth movements pooled for all teeth was significantly (*p* < 0.001) smaller in the control group compared to the study group for all directions despite vertical translational movements (along the Z‑axis, *p* = 0.646).

#### Tooth movements along and around the X-axis

**Fig. 4:**
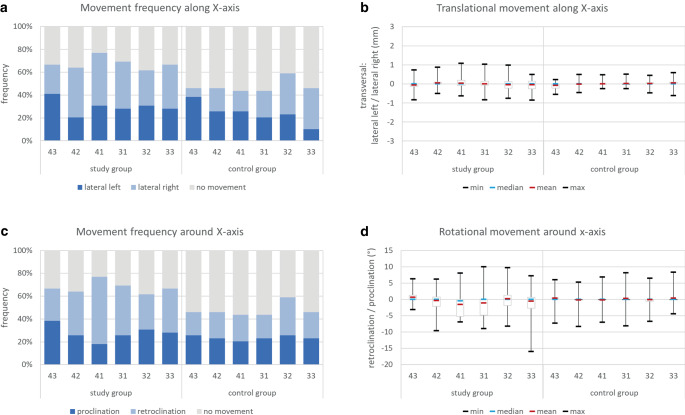
Tooth movements along (**a**, **b**) and around (**c**, **d**) the X‑axis. **a** Frequency of translational tooth movements, **b** amount of translational tooth movements in mm, **c** frequency of rotational tooth movements, and **d** amount of rotational tooth movements in ° Zahnbewegungen entlang der (**a**, **b**) und um die (**c**, **d**) X‑Achse. **a** Häufigkeit translatorischer Zahnbewegungen, **b** Ausmaß translatorischer Zahnbewegungen in mm, **c** Häufigkeit rotatorischer Zahnbewegungen, **d** Ausmaß rotatorischer Zahnbewegungen in °

For translational movements along the X‑axis (Fig. 4a; Table 1), the frequency of right and left tooth translation was nearly equally distributed among the different teeth in the study group, while in the control group, a diametrical prevalence at the canines was seen. The mean and median amount of lateral translational movements ranged between 0 and 0.1 mm, while maximum values ranged between 0.5 and 1.1 mm (Fig. 4b; Table 1).

**Table 1:** Absolute number of stable and affected patients per movement direction and group; mean values, standard deviations (SD), minimum, median, and maximum values as well as 25th and 75th percentiles of tooth movement measured by three-dimensional superimposition per tooth in the study and control group Absolute Anzahl stabiler und betroffener Patienten pro Bewegungsrichtung und Gruppe; Mittelwerte, Standardabweichungen (SD), Minima, Mediane und Maxima sowie 25. und 75. Perzentile der mittels 3‑D-Überlagerung gemessenen Zahnbewegungen pro Zahn der Studien- und der Kontrollgruppe

		Study group						Control group					
		43	42	41	31	32	33	43	42	41	31	32	33
Translational along X‑axis [mm]	Stable [*n*]	13	14	9	12	15	13	21	21	22	22	16	21
	Lateral right (+) [*n*]	10	17	18	11	12	15	3	8	7	9	14	14
	Lateral left (−) [*n*]	16	8	12	16	12	11	15	10	10	8	9	4
	Mean	−0.07	0.04	0.03	−0.01	−0.06	−0.05	−0.09	−0.02	−0.01	0.02	0	0.04
	SD	0.33	0.25	0.33	0.34	0.35	0.33	0.18	0.16	0.12	0.13	0.19	0.20
	Minimum	−0.84	−0.51	−0.64	−0.85	−0.77	−0.86	−0.56	−0.46	−0.26	−0.27	−0.49	−0.62
	25th	−0.12	0	−0.08	−0.07	−0.22	−0.26	−0.23	0	0	0	0	0
	Median	0	0	0	0	0	0	0	0	0	0	0	0
	75th	0	0.09	0.19	0.14	0.13	0.14	0	0	0	0	0.07	0.10
	Maximum	0.72	0.87	1.07	1.03	0.97	0.49	0.21	0.48	0.47	0.50	0.44	0.59
Rotational around X‑axis [°]	Stable [*n*]	13	14	9	12	15	13	21	21	22	22	16	21
	Proclination (+) [*n*]	15	10	7	10	12	11	10	9	8	9	10	9
	Retroclination (−) [*n*]	11	15	23	17	12	15	8	9	9	8	13	9
	Mean	0.62	−0.45	−1.61	−1.14	0.15	−0.62	0.34	−0.22	−0.19	0.20	−0.13	0.31
	SD	2.31	3.52	3.88	4.42	3.92	4.01	2.36	2.28	2.85	2.88	2.48	2.23
	Minimum	−3.16	−9.60	−7.01	−8.97	−8.24	−15.99	−7.32	−8.36	−7.09	−8.19	−6.78	−4.52
	25th	−0.12	−2.14	−5.21	−4.78	−1.84	−2.72	0	0	0	0	−0.53	0
	Median	0	0	−0.50	0	0	0	0	0	0	0	0	0
	75th	1.28	0.72	0	0.12	1.30	0.66	0.47	0	0	0	0	0
	Maximum	6.27	6.16	7.97	9.91	9.62	7.13	5.92	5.22	6.76	8.10	6.42	8.26
Translational along Y‑axis [mm]	Stable [*n*]	13	14	9	12	15	13	21	21	22	22	16	21
	Protrusion (+) [*n*]	6	11	16	13	11	17	3	5	7	8	7	7
	Retrusion (−) [*n*]	20	14	14	14	13	9	15	13	10	9	16	11
	Mean	−0.18	−0.08	−0.02	−0.03	0.03	0.16	−0.18	−0.13	−0.06	−0.07	−0.14	−0.09
	SD	0.4	0.50	0.58	0.64	0.62	0.57	0.38	0.37	0.31	0.34	0.33	0.32
	Minimum	−1.44	−1.60	−1.31	−1.34	−1.13	−1.11	−1.23	−1.20	−0.87	−0.96	−0.77	−0.81
	25th	−0.35	−0.31	−0.33	−0.27	−0.28	0	−0.43	−0.25	−0.03	0	−0.43	−0.17
	Median	−0.03	0	0	0	0	0	0	0	0	0	0	0
	75th	0	0.07	0.45	0.39	0.25	0.47	0	0	0	0	0	0
	Maximum	0.75	0.99	1.43	1.93	2.33	2.46	0.72	0.63	0.65	0.71	0.69	0.61
Rotational around Y‑axis [°]	Stable [*n*]	13	14	9	12	15	13	21	21	22	22	16	21
	Lateral right (+) [*n*]	15	17	19	17	12	1	4	7	9	7	12	4
	Lateral left (−) [*n*]	11	8	11	10	12	25	14	11	8	10	11	14
	Mean	−0.30	0.34	0.64	0.40	0.10	−2.87	−0.06	0.02	0.10	−0.36	0.06	−0.75
	SD	3.01	2.17	2.69	2.21	2.22	4.08	1.22	1.64	0.76	1.57	1.68	2.10
	Minimum	−10.78	−4.80	−8.34	−6.01	−5.32	−15.57	−1.80	−3.50	−1.54	−7.88	−3.76	−6.54
	25th	−0.15	0	−0.26	−0.10	−0.92	−4.49	−0.66	−0.32	0	−0.11	−0.12	−1.60
	Median	0	0	0	0	0	−1.23	0	0	0	0	0	0
	75th	0.71	1.31	2.51	1.39	0.56	0	0	0	0	0	0.45	0
	Maximum	5.14	5.63	4.82	5.66	6.46	1.61	3.84	6.17	2.05	1.63	5.08	3.15
Translational along Z‑axis [mm]	Stable [*n*]	13	14	9	12	15	13	21	21	22	22	16	21
	Extrusion (+) [*n*]	22	22	25	21	22	17	14	15	16	15	21	16
	Intrusion (−) [*n*]	4	3	5	6	2	9	4	3	1	2	2	2
	Mean	0.28	0.34	0.32	0.38	0.40	0.23	0.28	0.33	0.37	0.32	0.38	0.25
	SD	0.42	0.51	0.49	0.52	0.51	0.53	0.52	0.56	0.57	0.55	0.50	0.43
	Minimum	−0.57	−0.66	−0.75	−0.47	−0.28	−0.65	−0.95	−0.64	−0.74	−0.62	−0.39	−0.42
	25th	0	0	0	0	0	0	0	0	0	0	0	0
	Median	0.07	0.12	0.18	0.20	0.12	0	0	0	0	0	0.26	0
	75th	0.57	0.62	0.66	0.82	0.69	0.49	0.73	0.73	0.80	0.78	0.69	0.44
	Maximum	1.41	1.80	1.51	1.79	1.58	1.49	1.35	1.72	1.68	1.67	1.67	1.52
Rotational around Z‑axis [°]	Stable [*n*]	13	14	9	12	15	13	21	21	22	22	16	21
	ccw (+) [*n*]	12	11	16	12	16	9	8	5	7	6	12	8
	cw (−) [*n*]	14	14	14	15	8	17	10	12	10	11	11	10
	Mean	−0.30	−0.44	−0.19	0.05	−0.85	−0.82	0.01	−0.33	−0.19	−0.27	0.09	−0.15
	SD	3.01	2.03	2.29	2.33	2.41	3.37	1.68	1.05	1.14	1.25	1.67	2.05
	Minimum	−10.78	−5.10	−6.32	−4.58	−7.66	−13.18	−4.49	−3.21	−4.35	−4.50	−3.67	−6.27
	25th	−0.15	−1.64	−1.41	−1.20	−2.34	−1.80	−0.03	−0.49	−0.12	−0.31	−0.15	−0.38
	Median	0	0	0	0	0	0	0	0	0	0	0	0
	75th	0.71	0.46	1.29	1.33	0	0	0	0	0	0	0.54	0
	Maximum	5.14	4.65	5.57	7.04	6.44	7.25	4.49	2.38	2.37	2.66	4.14	7.04

*mm* millimeter, *ccw* counterclockwise, *cw* clockwise

For rotational movements around the X‑axis (Fig. 4c; Table 1), the central incisors of the study group showed a higher frequency for retroclination, while in the control group a more homogenous distribution of pro- and retroclination prevailed. In the study group, the average retroclination of the central incisors was greater than 1° and outliers up to 16° of retroclination and 9.6° of proclination were seen. In the control group, mean and median amount of rotational movements ranged thoroughly around zero, while also outliers in both directions were visible (Fig. 4d; Table 1).

#### Tooth movements along and around the Y-axis

**Fig. 5:**
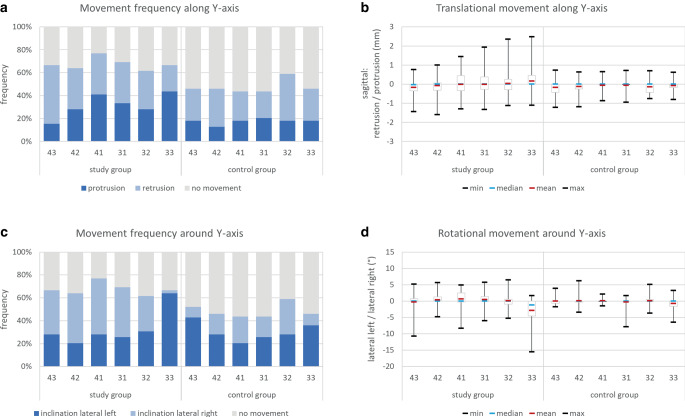
Tooth movements along (**a**, **b**) and around (**c**, **d**) the Y‑axis. **a** Frequency of translational tooth movements, **b** amount of translational tooth movements in mm, **c** frequency of rotational tooth movements, and **d** amount of rotational tooth movements in ° Zahnbewegungen entlang (**a**, **b**) und um die (**c**, **d**) Y‑Achse. **a** Häufigkeit translatorischer Zahnbewegungen, **b** Ausmaß translatorischer Zahnbewegungen in mm, **c** Häufigkeit rotatorischer Zahnbewegungen, **d** Ausmaß rotatorischer Zahnbewegungen in °

For translational movements along the Y‑axis (Fig. 5a; Table 1), an opposite movement direction was seen for the canines in the study group: the right canine showed predominantly retrusive movements, while the left canine was more often affected by protrusion. This pattern is also visible in the means, interquartile ranges, and maximum protrusive values of the study group, and can be brought into agreement with the so-called twist-effect. Despite the similarity of the mean values in the study and control groups which ranged between 0 and 0.2 mm, no characteristic movement pattern was detectable for the canines in the control group (Fig. 5b; Table 1).

For rotational movements around the Y‑axis (Fig. 5c; Table 1), no clear frequency distribution was visible. While inclinations to the right were more often seen for teeth 31–43 in the study group, tooth 33 showed a markedly larger prevalence for inclinations to the left. This is also confirmed by the amount of inclination (Fig. 5d; Table 1): the left canine presented an average inclination to the left of 2.9° and a maximum inclination of 15.6° in the same direction. For the other teeth in the study group, mean and median amounts ranged between 0 and 0.6° in both directions. In the control group, the mean and median values ranged between 0 and 0.8° of inclination to the left. The latter was also seen for the left canine.

#### Tooth movements along and around the Z-axis

**Fig. 6:**
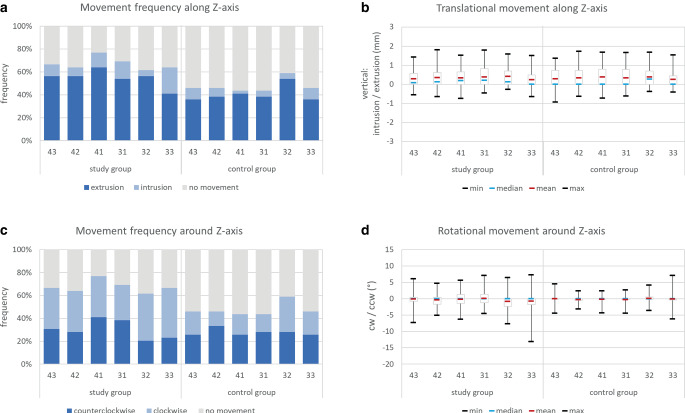
Tooth movements along (**a**, **b**) and around (**c**, **d**) the Z‑axis. **a** Frequency of translational tooth movements, **b** amount of translational tooth movements in mm, **c** frequency of rotational tooth movements, and **d** amount of rotational tooth movements in ° Zahnbewegungen entlang (**a**, **b**) und um die (**c**, **d**) Z‑Achse. **a** Häufigkeit translatorischer Zahnbewegungen, **b** Ausmaß translatorischer Zahnbewegungen in mm, **c** Häufigkeit rotatorischer Zahnbewegungen, **d** Ausmaß rotatorischer Zahnbewegungen in °

For translational movements along the Z‑axis (Fig. 6a; Table 1), a much higher frequency of extrusive movements compared to intrusive movements was seen in both groups. The mean amount of extrusion ranged between 0.2 and 0.4 mm in both groups, where the maximum extrusive values were also comparable between the groups and ranged between 1.4 and 1.8 mm. Intrusive outliers were also equal in both groups (0.3–0.7 mm) and interestingly, the largest value was found for tooth 43 of the control group with 0.9 mm intrusion (Fig. 6b; Table 1).

For rotational movements around the Z‑axis (Fig. 6c; Table 1), no distinct movement pattern (clockwise/counterclockwise) was seen for the study or for the control group. The mean amount of movement around the Z‑axis was around 0 for nearly all teeth in both groups, while the teeth 32 and 33 of the study group showed a more pronounced tendency for clockwise rotation reflected by the means (0.8°) and interquartile ranges. Maximum outliers ranged from 13.2° clockwise to 7.3° counterclockwise rotation in the study group and also in the control group, outliers in both direction were seen (Fig. 6d; Table 1).

### Possible predisposing factors

At all timepoints, the control group had a larger intercanine distance compared to the study group, which was found to be statistically significant at T1 (study: 26.36 mm, control: 27.27 mm, *p* = 0.019) and T2 (study: 26.39 mm, control: 27.20 mm, *p* = 0.037). The intercanine expansion during active orthodontic treatment was slightly larger in the study group, which was, however, not statistically significant. All further metric pretreatment- and treatment-related factors did not reach statistical significance (Table 2). The same was true for pretreatment habits as well as the variation in interincisal relationships (Table 3).

**Table 2:** Mean values and standard deviations (SD) of metric pretreatment- and treatment-related variables in the study and control groups Mittelwerte und Standardabweichungen (SD) der metrischen prätherapeutischen und therapeutischen Variablen der Studien- und der Kontrollgruppe

	Study group		Control group		*p*-value
	Mean	SD	Mean	SD	
Age at T0 [years]	12.82	1.92	12.22	1.87	0.160
Age at T1 [years]	15.83	1.82	15.39	1.58	0.210
Age at T2 [years]	17.89	1.73	17.47	1.54	0.481
Duration of treatment [years]	3.01	1.07	3.17	1.23	0.664
Duration of retention phase [years]	2.05	0.38	2.08	0.32	0.964
ML/NSL [°]	34.99	5.37	32.58	4.40	0.266
IiL/NB [°]	23.17	6.05	23.76	6.21	0.557
Ii-APo [mm]	0.99	2.39	0.74	2.22	0.443
Overjet at T0 [mm]	3.74	1.84	3.46	1.75	0.485
Overjet at T1 [mm]	2.14	1.04	2.01	0.79	0.835
Overjet at T2 [mm]	2.31	1.22	2.17	0.78	0.773
Overjet reduction during treatment (T0–T1) [mm]	1.60	1.94	1.45	1.92	0.595
Intercanine distance T0 [mm]	25.39	2.56	26.36	2.15	0.257
Intercanine distance T1 [mm]	26.36	1.87	27.27	1.40	0.019*
Intercanine distance T2 [mm]	26.39	1.90	27.20	1.25	0.037*
Expansion of intercanine distance during treatment (T0–T1) [mm]	0.97	2.30	0.91	1.70	0.663

*ML/NSL* mandibular plane angle, *IiL/NB* incisor inclination angle, *Ii-APo* incisor inclination relative to A‑Pogonion line, *mm* millimeters

*Statistically significant difference

**Table 3:** Absolute and relative frequency of premolar extractions, pretreatment habits, and interincisal relationship for the study and control groups Absolute und relative Häufigkeit von Prämolarenextraktionen, prätherapeutischen Habits und interinzisaler Relation der Studien- und der Kontrollgruppe

	Study group		Control group		*p*-value
	*n*	%	*n*	%	
*Premolars extracted during treatment*					
Yes	6	15.4	14	35.9	0.038*
No	33	84.6	25	64.1	
*Habits pretreatment*					
Yes	23	58.9	20	51.3	0.495
No	16	41.1	19	48.7	
*Interincisal relationship T0*					
Interincisal contact	31	79.5	34	87.2	0.362
Overlap without contact	7	17.9	5	12.8	0.530
Open bite	1	2.6	0	0.0	1
*Interincisal relationship T1*					
Interincisal contact	31	79.5	29	74.4	0.591
Overlap without contact	8	20.5	10	25.6	0.591
Open bite	0	0.0	0	0.0	1
*Interincisal relationship T2*					
Interincisal contact	30	76.9	32	82.1	0.575
Overlap without contact	8	20.5	5	12.8	0.362
Open bite	1	2.6	2	5.1	1

*Statistically significant difference

All teeth of the study group except tooth 32 were more often affected by detachment of bonding sites between T1 and T2. This issue only reached statistical significance in tooth 41 (*p* ≤ 0.001, Table 4). Wire breakages were very seldom in both groups (study group: *n* = 2, control group: *n* = 1) and, therefore, neglectable.

**Table 4:** Absolute and relative frequency of bonding site detachments during supervised retention period for the study and control group Absolute und relative Häufigkeit von Klebestellenverlusten während der überwachten Retentionszeit der Studien- und der Kontrollgruppe

Detachments of bonding sites (T1–T2)	Study group		Control group		*p*-value
	n	%	n	%	
33	6	15.4	2	5.1	0.263
32	3	7.7	3	7.7	1
31	9	23.1	3	7.7	0.600
41	17	43.6	0	0.0	<0.001***
42	7	17.9	6	15.4	1
43	5	12.8	1	2.6	0.200

***Statistically significant difference

### Classification of tooth movements based on rotational severity thresholds

**Fig. 7:**
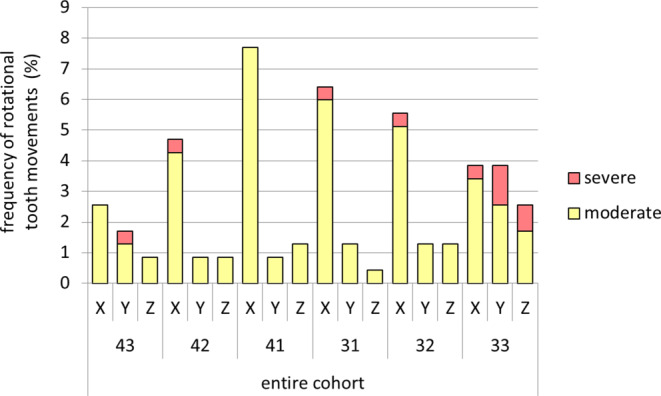
Distribution of posttreatment tooth movement severity on tooth level, based on the digitally measured rotational movements for the entire patient cohort. Classification of severity according to the thresholds used by Wolf et al. [34] Verteilung des Schweregrades posttherapeutischer Zahnbewegungen auf Zahnebene, basierend auf den digital vermessenen rotatorischen Bewegungen für die gesamte Patientenkohorte. Die Klassifikation des Schweregrades erfolgte gemäß der von Wolf et al. [34] verwendeten Grenzwerte

Applying the rotational severity thresholds introduced by Wolf et al. [34] to the present study population, the majority of the study group (64.1%) showed moderate rotational movements of ≥ 5° to ≤ 9°. Severe movements >9° were observed in 12.8%, while 23.1% of the study group patients presented movements between 0 and 5° which would be considered as a stable tooth position. In the control group, the majority of patients (71.8%) fell into the stable category, whereas 28.2% showed moderate rotational movements. None of the control group patients showed movements larger than 9° (Table 5). Allocating the entire patient cohort (study and control group) into to the severity categories, the incisors were most often affected by rotational movements around the X‑axis (proclination/retroclination), while the left canine presented a relatively equal distribution among the axes and fell also most often into the severe category (Fig. 7).

**Table 5:** Distribution of posttreatment tooth movement severity on the patient level, based on the digitally measured rotational movements among all axes of study group, control group, and the entire patient cohort. Classification of severity according to the thresholds used by Wolf et al. [34] Verteilung des Schweregrades posttherapeutischer Zahnbewegungen auf der Patientenebene, basierend auf den digital vermessenen rotatorischen Bewegungen über alle Achsen für die Studiengruppe, die Kontrollgruppe und die gesamte Patientenkohorte. Die Klassifikation des Schweregrades erfolgte gemäß der von Wolf et al. [34] verwendeten Grenzwerte

Severity classification	Study group		Control group		Entire cohort	
	*n*	%	*n*	%	*n*	%
Stable (< 5°)	9	23.1	28	71.8	37	47.4
Moderate (≥ 5° to ≤ 9°)	25	64.1	11	28.2	36	46.2
Severe (> 9°)	5	12.8	0	0.0	5	6.4

**Fig. 8:**
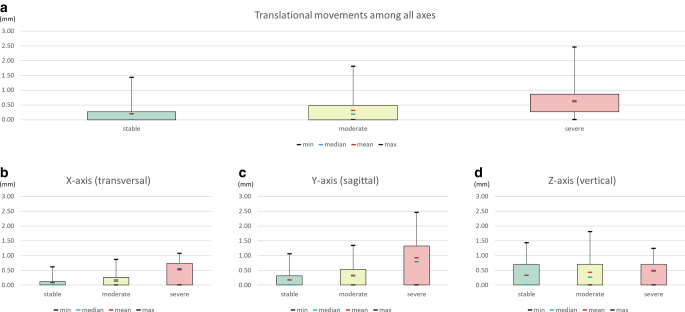
Amount of translational movements in mm of the total study cohort (*n* = 78) based on the severity categories according to Wolf et al. [34], depicted in *green* (stable), *yellow* (moderate), and *red* (severe). **a** Translational tooth movements pooled for all axes, **b** transversal movements, **c** sagittal movements, and **d** vertical movements Ausmaß translatorischer Bewegungen in mm der kompletten Studienkohorte (*n* = 78) basierend auf der Schweregrad-Kategorisierung nach Wolf et al. [34], dargestellt in *grün* (stabil), *gelb* (moderat) und *rot* (schwer). **a** Translatorische Zahnbewegungen aggregiert für alle Achsen, **b** transversale Bewegungen, **c** sagittale Bewegungen, **d** vertikale Bewegungen

In the entire patient cohort (study and control group), the mean translational movement pooled for all axes was 0.19 ± 0.32 mm for patients in the stable category, 0.31 ± 0.38 mm for those in the moderate category and 0.64 ± 0.5 mm for those in the severe category (Fig. 8a). Looking at the different axes, the mean amount of translational movements along the X‑ and Y‑axis coincided with the severity classification based on the rotational movements (Fig. 8b and c), while for the Z‑axis indicating vertical translational movements (extrusion/intrusion) equal mean values in all severity categories were seen (Fig. 8d). Interestingly, outliers up to 1.1 mm of protrusion/retrusion in the Y‑axis were observed even in the stable category (Fig. 8c).

## Discussion

To our knowledge, besides the work of Wolf et al. [34], this is the second study which measured translational and rotational tooth movements caused by fixed retainers in the lower jaw using 3D superimposition of digitized models. In accordance with previous studies and case reports/case series which observed different movement patterns [17, 28, 34], the measurements of the present study confirmed the twist-effect along the Y‑axis in the largest patient sample up to date. In the present study group, tooth 33 was more often protruded and tooth 43 more often retruded—this corresponds to the findings of Kucera and Marek [17], who found the same direction in 89.5% of their patients affected by the twist-effect. As possible reasons, the structure of the flexible spiral wire retainer and its winding/unwinding direction has been discussed in literature [17, 34]. Another hypothesis is the order of teeth during the bonding process, which could be influenced by the position of the practitioner which is usually on the right side of the patient and even by the right- or left-handedness of the practitioner. In addition, it cannot be ruled out that occlusal forces generated by mandibular growth changes may have contributed to the unwanted translational and rotational tooth movements. However, the detailed underlying mechanisms are still unknown. Furthermore, the timing of unwanted tooth movements still remains unclear. In one of the first studies addressing this complication, the majority of events occurred within the first 6 years in retention [17], so it could be questioned if the observation period of 2 years chosen in the present study was appropriate. Looking at the literature, comparable studies present follow-up periods reaching from 6 months to 3 years [11, 12, 34], so the present follow-up period seems acceptable.

Corresponding to previous results [12, 13, 34], vertical translational movements along the Z‑axis (extrusion/intrusion) showed a nearly equal prevalence and amount in the study as well as in the control group. This underlines the assumption that extrusive movements are not reflecting a retainer complication in terms of unwanted tooth movements but they express tendencies of settling after multibracket appliance debonding, slight relapse of deep bite or individual vertical growth. Studies investigating the deep bite relapse approximately 2 years after active treatment found an increase in overbite of 0.5–1 mm [3, 4, 19]. Supposing that the increase in overbite is the sum of extrusive movements of the upper and lower anterior teeth, the average values found in the present patient cohort (0.2–0.4 mm in the lower jaw, 0.3–0.5 mm in the upper jaw [13]) correspond well to the increase in overbite mentioned above [12, 13, 34]. As longitudinal growth studies revealed that the curve of Spee remains relatively stable during late adolescence and early adulthood [6, 9, 21], vertical growth seems not to be the main reason for the extrusive movements found in the present and corresponding studies [12, 34].

Applying the severity categories based on the arbitrary rotational movement thresholds introduced by Wolf et al. [34] to the present patient cohort, good agreement of the severity of the rotations with the translational means, third quartiles and maximum values were seen for the X‑ and Y‑axis (Fig. 8b and c). Nevertheless, even in the patients in the stable category, maximum tooth movements greater than 1 mm of protrusion/retrusion (Y-axis, Fig. 8c) were observed, which has to be considered clinically relevant. Therefore, the authors of the present study would suggest setting clinically relevant thresholds for translational movements between 0.5 and 1.0 mm for transverse and sagittal tooth movements. As a vision for the future, intraoral scans obtained during the supervised retention phase could be superimposed with the digital model at the debonding date, and a color-coded distance map (heatmap) indicating movements between 0.5 and 1.0 mm in the sagittal or transverse dimension could sensitize the practitioner for closer supervision of the patient, debonding of the retainer for spontaneous recovery [14] or even orthodontic retreatment, if necessary.

Regarding the possible predisposing factors for unwanted tooth movements, none of the factors identified in previous studies [12, 17, 34] could be confirmed in the present sample. Nevertheless, the greater intercanine expansion found in the study group could be a hint towards the findings of Wolf et al. [34] that greater expansion of the intercanine distance could enhance the risk of unwanted tooth movements. The fact that nearly all teeth of the present study group showed bonding site detachments more frequently than the control subjects although without reaching statistical significance could also sensitize the practitioner for a more critical supervision of patients with more retainer repairs.

The present study has some limitations. First, no sample size calculation was carried out a priori due to the retrospective study design and the limited number and heterogeneity of corresponding studies. Second, many patients were excluded from the study because of the strict inclusion criteria. A large number of patients had to be excluded because of damaged plaster casts which were not suitable for digital superimposition. This is a common problem of retrospective investigations, since the manufacturing of plaster casts was not mainly driven by the intention to reach quality standards high enough for digital superimposition in the future. Further, the storage of plaster casts over years especially at an university department archive can lead to damaged casts because many investigators went through the archive for different research or teaching purposes inevitably causing some accidents resulting in damaged plaster casts. In addition, many patients were excluded due to the required pretreatment class I malocclusion. This inclusion criterion was chosen because a preliminary study showed that patients with unwanted tooth movements presented an overbite with a lack of interincisal contact more often [12]. The exclusion of class II and III subjects was driven by the intention to rule out patients without interincisal contact due to the nature of their sagittal malocclusion. One could argue that an inclusion of class II and III patients in order to increase the total number of patients would have been beneficial. Third, the retainers were bonded by different orthodontists and residents of the department instead of one single operator. Nevertheless, a strict bonding protocol [37] was applied to keep the influence of this factor as small as possible. Fourth, screening of unwanted tooth movements was performed by visual inspection of T1 and T2 plaster casts, which is inevitably a subjective decision. The shortcomings of the visual inspection were demonstrated by applying the rotational severity thresholds according to Wolf et al. [34], showing that nearly one third of the control group without visible tooth movements fell in the moderately affected category presenting rotations of 5–9°. Nevertheless, the visual inspection of our patients is clinical practice in orthodontic treatment and supervision during the retention phase. Therefore, it seemed acceptable to use visual inspection added by consensus rounds for the screening of eligible study patients. Fifth, the digital superimposition in the lower jaw remains an unsolved problem due to the lack of anatomical structures which remain relatively stable over time [29]. However, corresponding literature reported best-fit superimpositions of lower jaws using molars [24, 34], molars and premolars [12, 14], or even molars and deciduous molars [33] for different research questions, so the use of premolars and molars in the present study seemed an acceptable compromise especially over the short time period of approximately 2 years. Nevertheless, one should keep in mind the limited accuracy of best-fit superimpositions of the lower jaw.

All in all, the still unknown etiology of unexpected tooth movements despite bonded lingual retainers in situ and the heterogeneity of results especially regarding possible risk factors underline the need for further research. The suggested translational tooth movement thresholds should ideally be validated in a larger patient cohort in the future.

## Conclusions

Translational and rotational movements in all three dimensions occurred despite bonded lingual retainers. Although the mean tooth movements were quite small (translation 0–0.4 mm, rotation 0–1.6°), large individual movements up to 2.5 mm protrusion and 16° retroclination were visible in the study group. A twist-effect with opposite movements of the canines along the Y‑axis could be confirmed. Patients with a larger intercanine distance after active treatment and those with more frequent retainer bonding site detachments could be at higher risk. Vertical extrusive movements seem to be no complication in sense of unwanted tooth movements, whereas sagittal and transverse movements of 0.5–1.0 mm visualized with color-coded distance maps of digitally superimposed casts during the retention time should sensitize the practitioner for further measures.

## Data Availability

The datasets supporting the conclusions of this article are available from the corresponding author on reasonable request.
